# Pulmonary Squamous Cell Carcinoma with Repeated Increases and Decreases in Size

**DOI:** 10.70352/scrj.cr.25-0067

**Published:** 2025-07-01

**Authors:** Yuji Nozaka, Yuji Ohtsuki, Yasushi Horie, Kengo Yasuda, Yuzo Takagi

**Affiliations:** 1 Department of General Thoracic Surgery, Tottori Prefectural Kousei Hospital, Kurayoshi, Tottori, Japan; 2 Department of Diagnostic Pathology, Tottori Prefectural Kousei Hospital, Kurayoshi, Tottori, Japan; 3 Department of Surgery, Division of General Thoracic Surgery, Breast and Endocrine Surgery, Faculty of Medicine, Tottori University, Yonago, Tottori, Japan

**Keywords:** lung cancer, squamous cell carcinoma, spontaneous regression, cholangitis

## Abstract

**INTRODUCTION:**

Spontaneous regression of cancer is rare. Although the underlying mechanism is unknown, immunological responses are thought to be involved.

**CASE PRESENTATION:**

An 82-year-old Japanese man presented with an incidental bile duct tumor and a dilated common bile duct noted on computed tomography of the abdomen. The scan also showed a 1.1 cm snowball-like shadow in the right lower lobe of the lung. Subsequently, he developed obstructive cholangitis and underwent endoscopic retrograde biliary drainage. Over 4 years of follow-up, the lung nodule repeatedly increased and decreased in size, and new nodules appeared and disappeared. A bronchoscopic biopsy was then performed to evaluate an enlarged nodule, which was consistent with squamous cell carcinoma. He underwent right lower lobectomy with ND1b lymph node dissection. The final diagnosis was pulmonary squamous cell carcinoma (pT1cNXM0).

**CONCLUSIONS:**

Immune response activation may have caused the observed tumor shrinkage. Careful follow-up is warranted owing to the possibility of regrowth.

## INTRODUCTION

Spontaneous regression of cancer is rare. Although the underlying mechanism is unknown, immunological responses are thought to be involved. We report a patient with a pulmonary squamous cell carcinoma that repeatedly increased and decreased in size, suggesting regression due to immunological response.

## CASE PRESENTATION

An 82-year-old Japanese man with a history of hypertension and benign prostate hypertrophy presented with an incidental bile duct tumor and dilated common bile duct noted on computed tomography of the abdomen. The scan also showed a 1.1 cm snowball-like shadow in the right lower lobe of the lung (S10). Subsequently, he developed obstructive cholangitis, and endoscopic retrograde bile duct drainage was performed because cholangiocarcinoma was also suspected. Stent replacement was performed 5 months later because of recurrent cholangitis caused by tube obstruction. Although his hepatobiliary enzyme concentrations then progressively decreased, computed tomography 15 months later showed worsening common bile duct and intrahepatic bile duct dilatation and enlargement of the bile duct tumor. Thereafter, no further cholangitis flare-ups or bile duct tumor enlargement occurred.

**Fig. 1:**
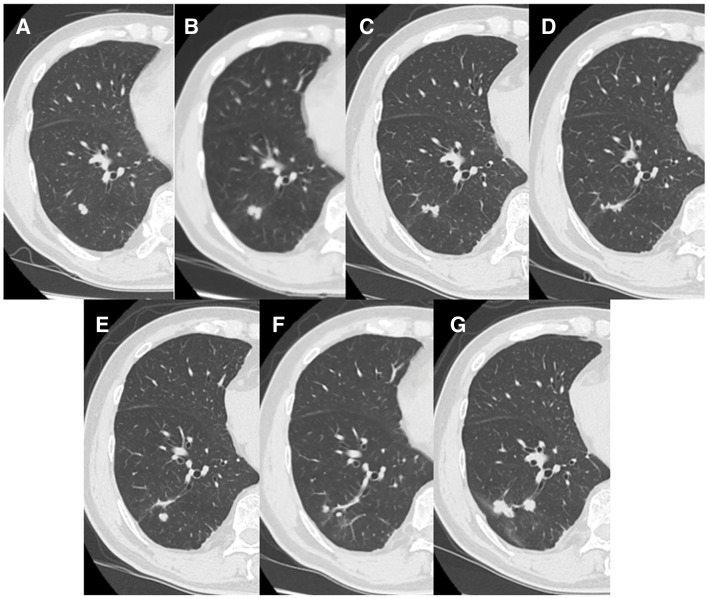
Chest computed tomography findings over time. At the time of initial detection, a snowball-shaped 1.1 × 0.7 cm nodule was seen in S10 of the right lower lobe (**A**). At the 11-month follow-up, it had mildly enlarged (**B**); however, it progressively decreased in size on the scans 4 months (**C**) and 9 months later (**D**). On the 25-month scan, its size had remained unchanged, but a new small nodule appeared in its vicinity (**E**). A year later, it had decreased in size again (**F**). At 49 months, it had enlarged to 3.2 × 0.8 cm (**G**).

The pulmonary lesion was followed over time with serial computed tomography. Over 4 years of follow-up, the lesion repeatedly increased and decreased in size, and new nodules appeared and disappeared (**Fig. 1**). He was then referred for surgical evaluation of an enlarged nodule.

**Fig. 2:**
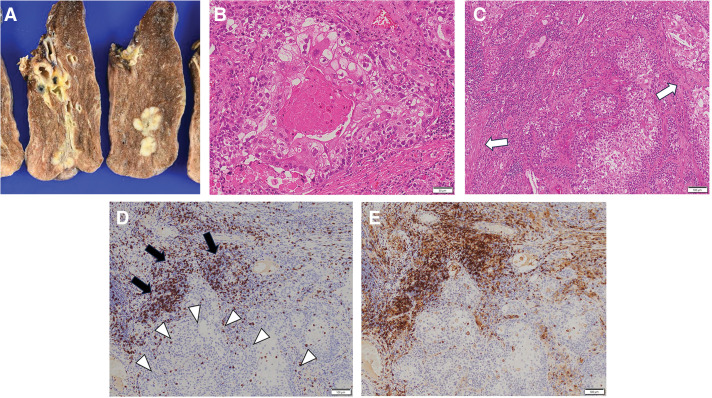
Pathological findings. Macroscopically, the tumor was 2.9 × 1.9 cm in size and solid (**A**). The tumor cells contained enlarged nuclei, were irregular in size, and exhibited central keratinization (**B**). Fibrosis (white arrows) and infiltration of lymphocytes were visualized within the tumor (**C**). Immunostaining showed dense infiltration of CD8- and CD4-positive T cells, mainly in the fibrotic areas (black arrows); their infiltration within the foci of tumor cells themselves was minimal (white arrowheads) (**D**, **E**).

A bronchoscopic biopsy was performed, which was consistent with squamous cell carcinoma. The patient then underwent right lower lobectomy with a preoperative diagnosis of cT2aN0M0 stage IB squamous cell carcinoma. Considering his advanced age and the tumor’s clinical stage, lymph node dissection was limited to ND1b. The #12l lymph node was submitted for intraoperative pathological examination and was negative. There was no adhesion in the thoracic cavity, nor any findings suggestive of inflammation, such as pleural effusion. Pathological findings are shown in **Fig. 2**. The tumor cells contained enlarged nuclei, were irregular in size, and exhibited central keratinization. Fibrosis and infiltration of lymphocytes were visualized within the tumor. Although immunostaining showed infiltration of many CD8- and CD4-positive T cells in fibrotic areas of the tumor, T cell invasion within the foci of tumor cells themselves was negligible. No lymph node metastases were observed. The final diagnosis was pulmonary keratinizing squamous cell carcinoma (pT1cNXM0). The disease has not recurred to date, and the patient is under outpatient observation.

## DISCUSSION

Spontaneous regression of cancer was defined by Everson in 1964 as “partial or complete disappearance of a malignant tumor in the absence of all treatment or in the presence of therapy which is considered inadequate to exert a significant influence on the growth of neoplastic disease,” a definition that is still widely used today.^1)^ The incidence of spontaneous regression is estimated to be 1 in 100000 cases.^2)^ The most common types of spontaneously regressing cancer are melanoma, renal cell carcinoma, neuroblastoma, and hematological cancers.^3,4)^ Lung cancers undergo spontaneous regression less frequently, possibly because they tend to be less immunogenic.^5)^

The mechanisms postulated to underlie spontaneous cancer regression include immunological responses, tumor inhibition by growth factors and/or cytokines, induction of differentiation, hormonal mechanisms, elimination of carcinogen, tumor necrosis and/or angiogenesis inhibition, psychological factors, apoptosis, and epigenetic mechanisms.^3,4,6)^ Among these, immunological responses are considered the most important.^7,8)^ Activation of the immune response to cancer triggered by infection, bronchoscopy, or surgery has been considered a factor in reports of spontaneous cancer regression dating back to 1976.^2,9)^

In our patient, a pulmonary nodule was discovered at the same time as the onset of cholangitis, which relapsed 5 months after endoscopic retrograde biliary drainage. Several previous reports have linked infections, including pneumonia, tuberculosis, and liver abscess, with lung cancer regression.^10–12)^ In our patient, the only apparent trigger of a systemic immune response contributing to tumor regression was cholangitis. However, his tumor decreased in size somewhat later than the onset of cholangitis; therefore, it is possible that cholangitis may not have played a role.

CD8-positive T cells play an important role in immunity against cancer. These cells recognize tumor-associated antigen peptides expressed by tumor cells and exert antitumor effects.^13)^ Moriyama et al. reported a high degree of CD8-positive T cell infiltration within spontaneously regressing foci of large-cell lung carcinoma and hypothesized that this infiltration may have caused the regression.^14)^

The surgical specimen from our patient also showed a high degree of CD8-positive T cell infiltration, mainly within fibrotic nests within the tumor, not within the tumor cell foci themselves. However, surgery was performed at the time of tumor regrowth, so the association of histological findings with tumor shrinkage or growth cannot be determined. Moreover, nests of necrosis and fibrosis can also be seen within typical squamous cell carcinoma, and their association with tumor shrinkage has not be determined. CD4-positive T cells and NK cells may also be responsible for immunity against cancer.^12,15,16)^ A study comparing histological findings before and after tumor regression is needed.

## CONCLUSIONS

We experienced a case of pulmonary squamous cell carcinoma that repeatedly increased and decreased in size. An immunological response may have been the mechanism underlying the observed regression and regrowth. The patient is currently being carefully followed in accordance with guidelines for management of pulmonary nodules,^17–19)^ because of the possibility of regrowth.

## ACKNOWLEDGMENTS

We thank Edanz (https://jp.edanz.com/ac) for editing a draft of this manuscript.

## DECLARATIONS

### Funding

Not applicable.

### Authors’ contributions

YN drafted the manuscript.

YT and YO performed a dedicated review and contributed to the discussion.

KY and YT performed the lobectomy and lymph node dissection.

YO and YH performed the pathological evaluation.

KY and YN performed follow-up after surgery.

All authors read and approved the final manuscript.

### Availability of data and materials

Not applicable.

### Ethics approval and consent to participate

This work does not require ethical considerations or approval. Informed consent to participate in this study was obtained from the patient.

### Consent for publication

The patient provided informed consent for publication of this case report and the accompanying images.

### Competing interests

The authors declare that they have no competing interests.
